# Supplementary material for: A high-resolution mRNA expression time course of embryonic development in zebrafish
Source: eLife. 2017 Nov 16;6:e30860. doi: 10.7554/eLife.30860 (PMC5690287; doi:10.7554/eLife.30860)
Supplement: Supplementary file 6. [file elife-30860-supp6.zip › biolayout-clusters-files/Cluster130-genes.html]

Cluster130


# Cluster130: Genes

| Ensembl ID | Gene Name | Chr | Start | End | Biotype |
| --- | --- | --- | --- | --- | --- |
| ENSDARG00000076933 | aldh1a3 | 7 | 9476221 | 9557990 | protein\_coding |
| ENSDARG00000034528 | chodl | 24 | 23869706 | 23883061 | protein\_coding |
| ENSDARG00000040087 | epb41l4a | 10 | 1717529 | 1799831 | protein\_coding |
| ENSDARG00000030932 | fgf10a | 21 | 19612178 | 19645986 | protein\_coding |
| ENSDARG00000025301 | gfap | 3 | 31660727 | 31672767 | protein\_coding |
| ENSDARG00000037960 | lrrc17 | 4 | 20420216 | 20435110 | protein\_coding |
| ENSDARG00000044155 | mafaa | 6 | 32714669 | 32718627 | protein\_coding |
| ENSDARG00000039095 | nkx2.3 | 13 | 40283325 | 40286027 | protein\_coding |
| ENSDARG00000103542 | vegfaa | 16 | 4458253 | 4488590 | protein\_coding |
| ENSDARG00000103443 | vwde | 15 | 34131202 | 34136439 | protein\_coding |
